# Improving the Leavening Effect of Ice like CO_2_ Gas Hydrates by Addition of Gelling Agents in Wheat Bread

**DOI:** 10.3390/gels9030223

**Published:** 2023-03-14

**Authors:** Shubhangi Srivastava, Ann Mary Kollemparembil, Viktoria Zettel, Antonio Delgado, Mario Jekle, Bernd Hitzmann

**Affiliations:** 1 Department of Process Analytics and Cereal Science, University of Hohenheim, 70599 Stuttgart, Germany; 2 Institute of Fluid Mechanics (LSTME), FAU Erlangen-Nüremberg, 91058 Erlangen, Germany; 3 German Engineering Research and Development Center LSTME Busan, Busan 46742, Republic of Korea; 4 Department of Plant-Based Foods, Institute of Food Science and Biotechnology, University of Hohenheim, 70599 Stuttgart, Germany

**Keywords:** gas hydrates, ice like, gelling agents, bread, egg white, ascorbic acid, rice flour

## Abstract

This article brings together the application of ice-like CO_2_ gas hydrates (GH) as a leavening agent in wheat bread along with the incorporation of some natural gelling agents or flour improvers into the bread to enhance the textural properties of the wheat bread. The gelling agents used for the study were ascorbic acid (AC), egg white (EW), and rice flour (RF). These gelling agents were added to the GH bread containing different amounts of GH (40, 60, and 70% GH). Moreover, a combination of these gelling agents in a wheat GH bread recipe was studied for each respective percentage of GH. The combinations of gelling agents used in the GH bread were as follows: (1) AC, (2) RF + EW, and (3) RF + EW + AC. The best combination of GH wheat bread was 70% GH + AC + EW + RF combination. The primary goal of this research is to gain a better understanding of the complex bread dough created by CO_2_ GH and its influence on product quality when certain gelling agents are added to the dough. Moreover, the prospect of managing and modifying wheat bread attributes by the use of CO_2_ GH with the addition of natural gelling agents has not yet been researched and is a fresh idea in the food industry.

## 1. Introduction

When water and low molecular weight gases are exposed to low temperature and high pressure conditions, gas hydrates (GH) develop. To stabilize the structure, suitable sized guest molecules are caged in hydrogen-bonded water molecules without any chemical interactions. The most prevalent GH guest molecules are ethane, nitrogen, and carbon dioxide [1,2,3,4].

Carbon dioxide has an effect on dough leavening. Yeast is widely utilized in the manufacture of bakery items such as bread. However, yeast leavening has a drawback. To begin with, yeast takes a long time to ferment before producing enough gas to make the dough expand to the proper size [5]. Furthermore, the dough storage space must be large enough to accommodate yeast leavening processes, and fermentation needs careful temperature and humidity control, which can be costly for bakeries. Eliminating fermentation and cutting proofing times would boost bread sector production. The basic idea for using CO_2_ GH as a leavening agent comes from the fact that CO_2_ GH can help to produce consistent bread products by removing yeast.

Producing bakery items is the process of transforming dough, which is primarily composed of flour and water (additional components such as sugar, egg, leavening agent, and other additions will vary depending on the product), into a food product [6]. Based on macroscopic observations, three key changes may be identified during baking. First and foremost, the creation of gases from leavening agents and water vaporization increases product thickness. Second, when the baked product weight decreases due to drying, an open porous structure arises. Finally, the baked product darkens due to sugar caramelization and the non-enzymatic Maillard process, which includes the interaction of reducing sugars and proteins [7]. The starch and protein composition of the combination determines the structure, rheology, and other physical features of baked goods, as well as additional components such as sugar and fat. The dough structure is formed as a result of the gelatinization and pasting of the starch and the coagulation of the egg protein during baking. In bread, gluten proteins form the fundamental structure. Sucrose changes the hydration properties of proteins and limits the quantity of water available to starch granules, resulting in starch gelatinization and protein denaturation. The ultimate volume of baked items is determined by the amount of gas produced and trapped in the dough as well as the amount of gas stabilized during the entire process [7,8]. For instance, the use of gelling agents or flour improvers can help. Ascorbic acid (AC) is essential to enhance the bread’s structure and ultimate loaf volume, as well as to boost dough strength. AC is converted into dehydro AC, which is an oxidizing agent that creates disulphide linkages to enhance the gluten network [9,10]. Based on the flour weight, 10–200 ppm of AC can be used for excellent dough processing with higher baked product quality [11]. Moreover, in commercial bakeries, AC is utilized as a flour enhancer with the primary goal of raising the dough and extending the bread’s shelf life. The dough is more elastic, stable, and able to expand quickly as a result of dehydro AC oxidizer’s assistance in the formation of connections between the glutinous chains that reinforce the gluten network. As a result, the dough will not rupture with inflation during baking [12,13]. Understanding the fundamental mechanics enables improved control of the bread crumb structure. In aerated systems, such as those in bakeries, egg white (EW) is frequently employed as a foaming agent [14] because of its outstanding reputation for producing a perfect baked structure [15,16]. The capacity of eggs to coagulate and create gels while being heated is another advantageous quality for baking systems [17]. Rice flour (RF) has higher starch, more viscosity, and damaged starch content [12]. Thus, the usage of the starch-based dough gives it a higher viscosity, and increases its gas-holding capacity and foam stability [18,19]. Apart from this, all the gelling agents or flour improvers mentioned above are natural, thus the addition of such ingredients would not pose a threat to human health [20,21,22].

However, the prospect of managing and modifying the quality of wheat bread through the use of CO_2_ GH and natural gelling agents in baking has not yet been researched and is a unique notion in the food industry. Customers’ appetite for clean-label meals is rapidly increasing, forcing the food sector to select creative and ecologically favorable raw materials. Nonetheless, essential understanding concerning the possible function of CO_2_ GH as leavening components along with natural gelling agents such as ascorbic acid, egg white, and rice flour in wheat bread baking and the consequent effect on the GH bread quality is still lacking. As a result, the purpose of this study is not only to present a summary of existing research in this area but also to elaborate on knowledge and understanding of underlying aspects that should be addressed while employing this CO_2_ GH technology to bake wheat bread.

## 2. Results and Discussion

**Figure 1:**
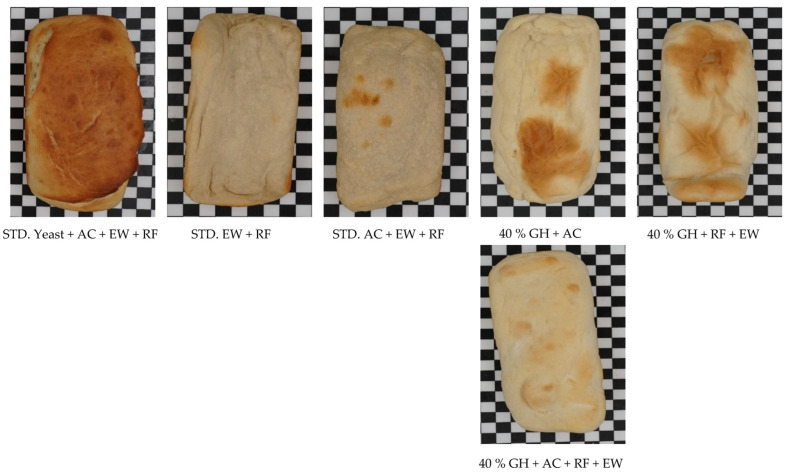
Pictures of GH bread (40% GH) with different gelling agents versus the standard bread. The effect of gelling agents along with GH improved the physical appearance of the bread.

Figure 1 shows the pictures of GH bread (40% GH) with different gelling agents versus standard bread. Standard wheat bread has a calorie value of 313 per 100 g and a protein content of 12% per 100 g. The addition of gelling agents, particularly EW and RF, influenced the calorie value of 640 per 100 g and with a protein content of 27% per 100 g of bread. Therefore, the gelling agents in the wheat bread particularly enhanced the nutritional aspect of the bread when the leavening was CO_2_ GH. It was discovered that the standard made with yeast, EW, and RF had significantly more browning than the other standard bread made only with EW and RF or the standard made with AC, EW, and RF. The reason might be attributed to the fact that yeast impacts bread color by producing secondary metabolites via several metabolic pathways via non-enzymatic chemical processes such as Maillard and caramelization [21,22,23], which generate brown colored chemicals during baking [24,25,26]. Moreover, it was found that the addition of 40% GH in the wheat bread along with gelling agents AC, EW, and RF further enhanced the physical appearance of the bread in comparison to the standards that were made alone with EW and RF or AC, EW, and RF combinations (Figure 1).

**Figure 2:**
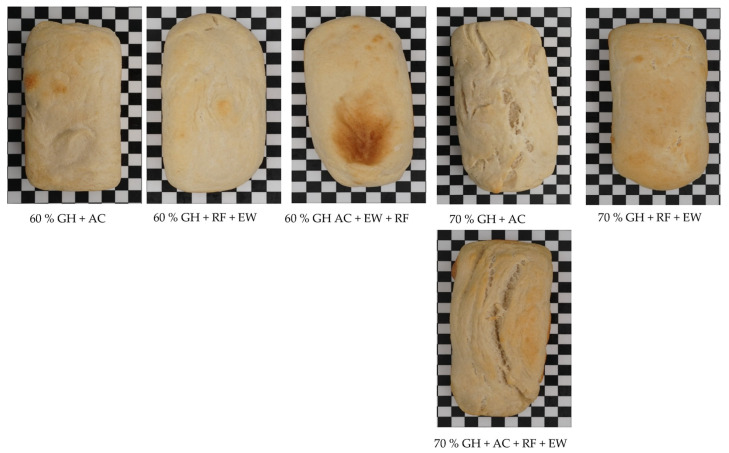
Pictures of GH bread (60 and 70% GH) with different gelling agents. The effect of gelling agents along with GH improved the physical appearance of the bread.

Figure 2 shows pictures of GH bread (60 and 70% GH) with different combinations of gelling agents (AC, RF + EW, and AC + RF + EW). It was clear from the pictures that with the increase in the percentage of the GH, the physical appearance of the GH bread with gelling agents improved further. The presence of CO_2_ GH in higher proportions leads to better involvement of the leavening effect via CO_2_ gas during baking. Moreover, it was found that the GH bread made with the combination of AC + RF + EW had a better appearance than the breads made with the combination of RF + EW or AC alone as gelling agents (Figure 2). The addition of RF as a gelling agent in the dough increased the viscosity of the dough, which increased the gas-upholding capacity of the dough. Then, further addition of the EW proteins as a gelling agent improved the gas cell stabilization of the GH dough system, and the presence of ascorbic acid also helped to stabilize the gluten protein network. Thus, it was quite evident from Figure 1 and Figure 2 that CO_2_ GH can be a source of a leavening agent if they are combined with a number of gelling agents or flour improvers for baking the bread.

Table 1 shows the characteristics of GH bread with different gelling agents with reference to the standard. The moisture content of the three reference standards prepared (Std. Yeast + EW + RF, Std. EW + RF, and Std. AC + EW + RF) lied in the range of 40–43%. Moreover, the GH wheat bread (40–70%) prepared with different combinations of gelling agents (AC, RF + EW, and AC + RF + EW) had a fairly acceptable range of moisture content 38–44%. The baking loss (BL) values for the standards ranged from 7–9%, while in the case of the GH bread with gelling agents, the BL values lied in the range of 6–17%. Higher BL was observed in the GH wheat bread prepared with 70% GH RF + EW (17.7%) and 70% GH AC + RF + EW (16.6%), respectively. The volume of the standard bread ranged from 802–1090 mL, while the GH bread made with gelling agents also had a fairly acceptable volume range of 519–989 mL. It was also found that an increase in the percentage of GH from 40 to 70% along with the gelling agents AC + RF + EW had a positive effect on the volume of the bread (Table 1). Thus, the GH bread made with gelling agents AC + RF + EW had a higher volume than the breads prepared with RF + EW or AC alone as an additive. Moreover, the specific volumes of the standard bread ranged from 1.1–2.1 mL/g, while the GH bread made with gelling agents also had a fairly acceptable volume range of 1.0–2.0 mL/g. Consequently, the GH bread made with gelling agents AC + RF + EW had a higher specific volume than the bread prepared with RF + EW or AC alone as an additive. As a result of the study’s findings, when GH breads are made with various combinations of gelling agents or flour improvers, the recipe has a better chance of improving when AC, RF, and EW are used. Carbon dioxide generation has the greatest impact on the volume of each loaf and the aerated cell structure of bread [24], and therefore, the ability of the dough to contain gas is a significant factor in determining bread quality during baking. Thus, a larger specific volume is achieved by having smaller gas cells and more evenly distributed CO_2_ gas cells, as more gas is retained in the dough [27,28,29].

**Figure 3:**
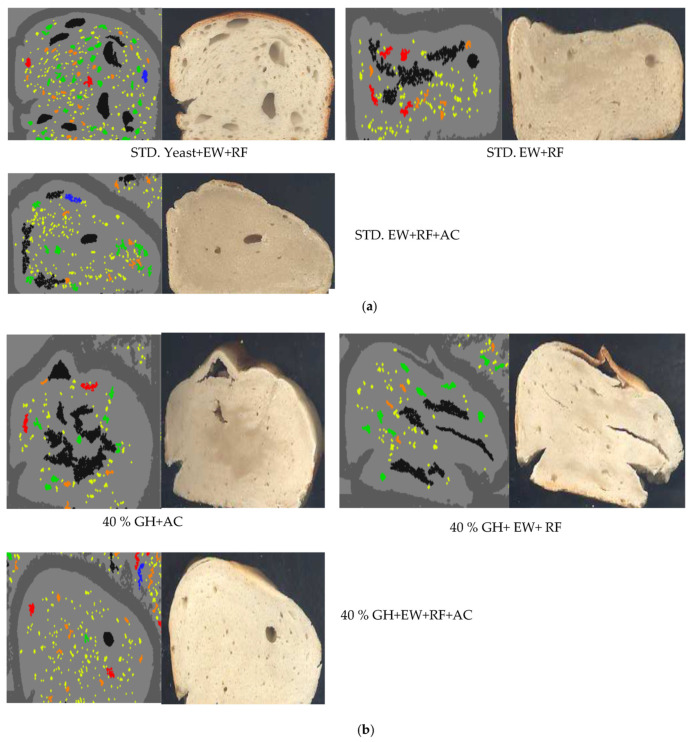
Cross sectional structures of GH bread with gelling agents in reference to gelling agents added to the standard breads. The addition of gelling agents along with GH improved pore distribution in the bread. The standards are shown in figure (**a**) with different combination of gelling agents with 40% GH in figure (**b**).

Figure 3 shows the cross sectional structures of GH bread with gelling agents in reference to different gelling agents added to standard breads. It was found that the cross sectional images of the standard yeast, EW, and RF, were better than those of the standards prepared with only gelling agents, such as EW + RF or AC + EW + RF. The absence of yeast in the standards (EW + RF or AC + EW + RF) further confirmed that (Figure 3a) the leavening is affected when there is no leavening agent present in the standard despite the presence of gelling agents (AC, EW, and RF). Furthermore, Figure 3b shows that when a trio of AC + EW + RF is combined with 40% GH, the bread produced has a far better appearance due to leavening effects. Moreover, the combinations 40% GH + AC or 40% GH + EW + RF need more amount of leavening so that they are better in textural properties.

**Figure 4:**
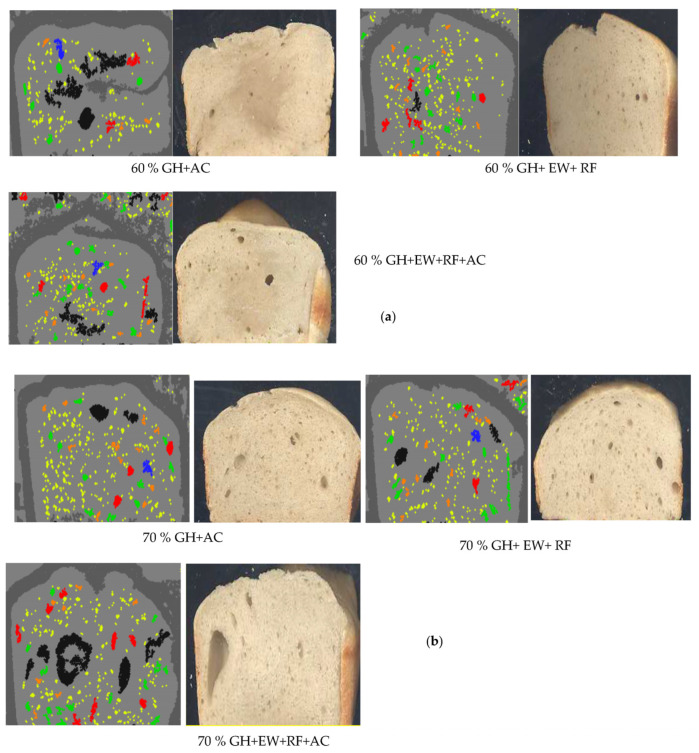
Cross sectional structures of GH bread (**a**) 60% GH and, (**b**) 70% GH with gelling agents. The addition of gelling agents along with an increased % of GH improved pore distribution in the bread.

Figure 4 shows the cross sectional structures of GH bread (60 and 70%) with gelling agents. Similarly, when a trio combination of AC + EW + RF was used as an additive along with 60 and 70% GH, the bread made was far better in appearance, with leavening effects depicted from the pores present, in comparison to the 60/70% GH + AC or 60/70% GH + EW + RF (Figure 4a,b) bread.

Table 2 shows the pore analysis of the GH wheat bread with different gelling agents in reference to the standard bread. The standard breads (Std. Yeast + EW + RF, Std. EW + RF, and Std. AC + EW + RF) had small pores roughly in the range of 82–92, medium pores in the range of 5.1–1.7, and little bigger pores in the range of 1.0–3.9, respectively. The number of small pores for the GH bread with gelling agents (AC, EW, and RF) ranged from 85–91, while medium pores were in the range of 8.3–10.7, and little bigger pores were in the range of 2.2–4.2. It was found that an increase in the amount of GH from 40 to 70% increased the amount of pores of different sizes, thereby confirming the percentage of leavening was more when a higher % of GH was used. Moreover, when the trio combination of gelling agents (AC + EW + RF) was used along with the GH, the bread had a higher number of pores in comparison to the bread made with a combination of EW + RF or AC alone. The percentage increase in the number of pores with GH and gelling agents further suggested that gelling agents play a vital role in further enhancing the overall structure of the bread.

**Figure 5:**
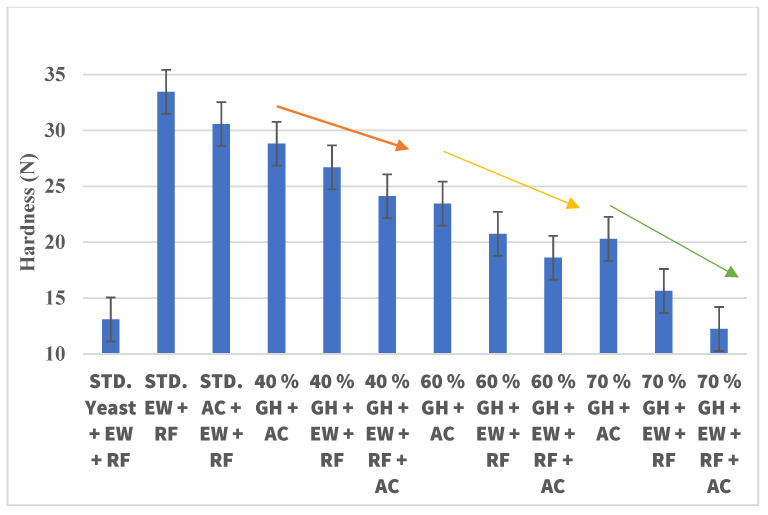
The hardness of the bread made with GH and different gelling agents. The value of hardness decreased when gelling agents were added to the bread.

Figure 5 shows the hardness of the bread made with GH and different gelling agents (AC, EW, and RF). The hardness of the standard bread made with yeast + EW + RF was recorded at 13.09 N, while the standard prepared with EW + RF had a hardness value of 33.45 N, and the standard bread made with AC + EW + RF was found to be 30.56 N. The standard error values of the samples lied in the range of ±4.8 to ±5.2. The samples of Std. Yeast + EW + RF were statistically different from the Std. EW + RF, and Std. AC + EW + RF samples with a *p*-value ≤ 0.05. There was also a significant difference in the samples of 70% GH + EW+ RF and 70% GH + EW + RF + AC breads compared with the Std. EW + RF and Std. + AC + EW + RF breads, respectively, with a *p* value ≤ 0.05. However, there was no significant difference between the samples of 40% GH + AC, 40% GH + EW + RF, or 40% GH + EW + RF breads. A similar trend was observed in 60% GH + AC, 60% GH + EW + RF, or 60% GH + EW + RF, and 70% GH + AC, 70% GH + EW + RF, or 70% GH + EW + RF breads, respectively. Moreover, no significant difference was also obtained in 70% GH + EW + RF + AC with respect to Std. yeast + EW + RF wheat breads, indicating that the breads made with the addition of 70% CO_2_ GH have hardness characteristics similar to those of original bread prepared with standard yeast, egg white, and rice flour. Therefore, it was clear that the amount of leavening agent played an important role in maintaining the texture of the bread, particularly the hardness. In spite of the addition of gelling agents to the standard, the hardness level of the bread was not up to the mark, as it was observed in the standards made with EW + RF or with AC + EW + RF.

The values of the hardness for the GH wheat bread (40–70% GH) ranged from 28.81–12.24 N. A clear grouping for a decrease in the hardness values was observed for each batch of GH, either with 40% (orange arrow), 60% (yellow arrow), or 70% (green arrow) GH along with the combinations of gelling agents (1) AC, (2) EW + RF, and (3) AC + EW + RF, respectively. The best combination of GH wheat bread with the least amount of hardness (12.24 N) was observed in a 70% GH + AC + EW + RF combination of bread. Thus, it can be said that to make bread with good textural features, the amount of leavening in the bread along with gelling agents in the dough system need to be balanced in adequate proportions.

One of the previous studies in this area of GH as a leavening agent showed that GH alone was not sufficient enough to make the bread softer in texture as it was in this case. The amount of hardness in the bread made with GH was a major concern when GH was added alone as a leavening agent [21]. The major strategy for resolving the issue raised in the previous research pertaining to the hardness of the GH bread is to blend certain functional gelling agents, to partially simulate wheat bread properties [21]. However, in this research study, a major change in the hardness levels of the GH bread was observed, which can be sufficiently explained by the presence of the gelling agents in the dough system along with the CO_2_ GH.

The improvement in the textural properties of the GH bread might be because of the RF, which has a higher viscosity; therefore, it helps in increasing the gas-holding capacity of the dough [7,18,19]. However, there is a catch while making the dough; if the amount of RF is not balanced with the amount of wheat flour, then there would be an excess of viscosity in the dough, which might again limit its ability. Therefore, in this study, a balance between the ratio of wheat flour and rice flour (90:10) was maintained, which resulted in an increase in the viscosity of the dough that was sufficient enough to expand the gas bubbles during baking, giving GH bread a proper specific volume. Moreover, the balance between CO_2_ release and crumb setting during baking determines the bread’s volume and the crumbs’ structure [12,19]. Hence, the EW proteins improved the gas cell stabilization of the GH dough system. Masure et al. [19] also found similar results, finding that with the addition of EW protein, gas cell stabilization improved, loaf volume increased, and a finer pore structure was found in the bread. Apart from the application of gelling agents, to get the desired GH wheat bread quality, the amount of water should also be properly regulated [30,31]. Low dough stability, overexpansion, and fragile crumb structure could be some of the causes of high water addition [7,19,32,33]. Therefore, this step was also crucial in getting better textural features with the GH bread with gelling agents. Furthermore, based on the results of the study, it was found that 70% GH+ AC + EW + RF wheat bread was found to be the best in terms of textural analysis, pore size analysis, and other physiochemical parameters. The justification for getting good results with the aforesaid mentioned GH wheat bread can be explained by the fact that the gas cells in the 70% GH + AC + RF + EW bread were properly stabilized, probably due to the presence of EW proteins, the RF viscosity factor, and lastly because of the ascorbic acid, which stabilized the gluten protein network in the GH dough. This resulted in a greater loaf volume and a finer structure for the bread.

## 3. Conclusions

One of the issues addressed in this research is the introduction of CO_2_ GH as a leavening agent combined with natural gelling agents for GH wheat bread. One of the main benefits of CO_2_ GH as a leavening agent would be that it is a desirable option for the continuous manufacture of yeast-free leavened dough, is chemical-free, and has no impact on the sensory qualities of the baked good. To improve the GH bread recipe, several gelling agents were used to improve the baking quality. There have been reports of contradictory findings for certain gelling agents or improvers. No one particular improver or additive resolves all the issues pertaining to bread baking. Hence, this study dealt with the addition of several gelling agents in combination, including ascorbic acid, rice flour, and egg white, which have the potential to be used as functional ingredients in the GH dough systems. More study and analysis are needed to better understand the difficulties and mechanisms at work, as well as to investigate the possibility of employing other improvers or multi-improvers to create greater quality bread prepared from the GH.

The greatest effects in CO_2_ GH wheat bread were promoted by a combination of rice flour, ascorbic acid, and egg white, followed by rice flour and egg white; these gelling agents strengthened the GH leavened wheat flour dough and thus showed their adequate properties to be used as gelling agents. Moreover, the GH wheat bread quality was positively affected by rice flour and egg white, which lead to GH bread with better specific volume and a softer crumb. Data from both pore analysis and textural bread properties drive on to conclude that ascorbic acid, rice flour, and egg white have good properties to be used as improvers in the baking of GH bread.

## 4. Materials and Methods

### 4.1. CO_2_ Gas Hydrates (GH) Production

The CO_2_ GH was created in a reactor installed at the Department of Process Analytics and Cereal Science, University of Hohenheim, Stuttgart, Germany, in conjunction with installation help from the Institute of Fluid Mechanics (LSTME), FAU Erlangen, Germany [3,21,22]. The CO_2_ GH was created by pouring 500 mL of distilled water mixed with natural amino acids as promoters into the reactor vessel, which was connected to the CO_2_ gas cylinder at a pressure of 37 bars and a temperature of 273.15 K. After the gas molecules have been dissolved at a low temperature, the GH synthesis begins with nucleation and lasts three hours [3,21].

### 4.2. Production of Wheat Bread with Gelling Agents

The wheat bread with gelling agents was made with 296.9 g of wheat flour (Type 550), 6 g of sugar, 6 g of salt, 0.05 g of ascorbic acid, and egg white of 28.89 g. The amount of rice flour added was 10% (29.69 g) of the amount of wheat flour used in a wheat bread recipe. The amount of water to be added was calculated for each percentage of GH (40, 60, and 70%) added. Some preliminary trials showed that each 10 g of GH included 2.5 g of CO_2_ and 7.5 g of water. To avoid watery dough, the quantity of water existing in the GH was removed from the total amount of water necessary for kneading.

To make the GH wheat bread, all the dry components (wheat flour, rice flour, and ascorbic acid) were weighed together, while the egg white and water were weighed individually. A minor change was made to the baking process to omit the proving stage of the wheat bread dough created with GH (40–70%). Before combining all of the dry components in the farinograph, the farinograph temperature was set to 42 °C. This step was required since the addition of GH decreases the temperature of the dough, and this modified step may overcome the low temperature of the GH dough, making it ideal for baking GH wheat bread. After one minute of mixing all the dry components in the farinograph, water, egg white, and GH were added and kneaded for three minutes. The kneaded dough (34 ℃) was weighed, divided, and rounded, followed by shaping and panning. The GH wheat bread was baked at 240 °C in a baking oven (Piccolo, Wachtel GmbH) for 22 min with 12 s of initial steaming.

### 4.3. Analysis of GH Wheat Bread

**Figure 6:**
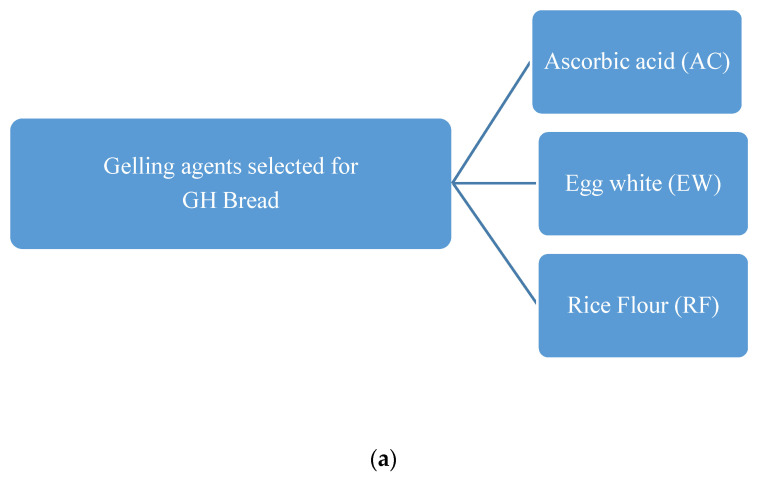

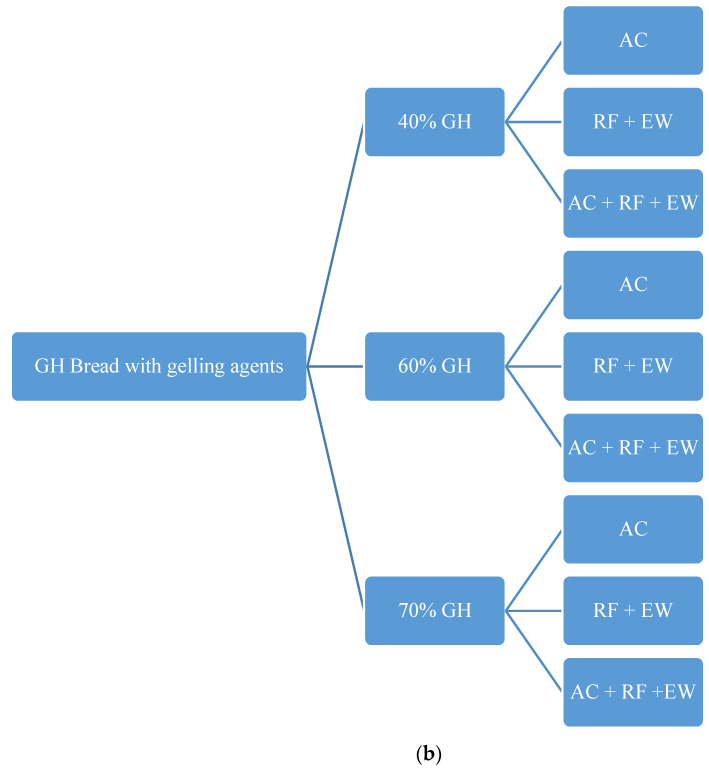
(**a**) The gelling agents selected for the study, and (**b**) variations carried out with gelling agents along with different percentages of GH (40–70%).

Figure 6a shows the gelling agents selected for the study, and Figure 6b shows the variations carried out with gelling agents along with different percentages of GH (40–70%). Different combinations of gelling agents were used in the GH wheat bread, as follows: (1) AC, (2) RF + EW, and (3) RF + EW + AC. In order to ensure an ideal comparison is made between the GH wheat bread and the standard bread, three different standards were made, taking into account the gelling agents that were to be added to the GH bread. The three standards prepared as a reference were: (1) Standard yeast + EW+ RF; (2) Standard EW + RF; and (3) Standard AC + EW+ RF respectively.

Moisture analysis, baking loss, volume analysis, pore analysis, and hardness analysis were used to measure the features of GH wheat bread. All the measurements for the bread were carried out after cooling the bread for one hour after baking. Moreover, all the analyses were carried out in triplicate, and the data were reported as means ± standard deviation. The statistical analysis was carried out using the Microsoft Excel add-on function XLSTAT, 2016. The ANOVA was used to determine the significant difference between the samples at a 95% confidence interval and a significance level of = 0.05.

The moisture of the GH wheat bread was measured using an infrared moisture analyzer. The baking loss was computed by dividing the weight of the bread by the weight of the dough, and then converting it to a percentage by subtracting one from the value obtained, and finally multiplying it by 100. The volume analysis of the GH bread was carried out using a volume analyzer (Stable Microsystems, VolScan Profiler 600). Before beginning the measurement of bread samples, a zero height calibration was carried out. An eye-safe laser tool was then used to scan vertically and measure the product’s contours as it spins. The specific volume was computed by dividing the volume of the bread obtained by the weight of the bread after baking. The pore size analysis was carried out by a self-constructed pore scanner (Hp scan jet 5590) connected to an inbuilt software (Gebäck analyse version 1.4) with oracle virtual tool box 6.1 developed by the University of Applied Sciences and Arts Ostwestfalen-Lippe, Lemgo, Germany. The hardness (N) of the bread was measured by means of a texture profile analyzer (TA-XT2, Stable Microsystems) with a P/36R 36 mm cylindrical probe.

## Figures and Tables

**Table 1:** The characteristics of GH bread with different gelling agents with reference to the standard.

	Standard			% of the GH								
	Std. Yeast + EW+ RF	Std. EW + RF	Std. AC + EW + RF	40 + AC	40 + RF + EW	40 + AC + RF + EW	60 + AC	60 + RF + EW	60 + AC + RF + EW	70 + AC	70 + RF + EW	70 + AC + RF + EW
MC (%)	43.3 ^a^± 2.0	38.7 ^a^ ± 2.0	40.9 ^a^ ± 2.1	38.7 ^a^ ± 2.1	39.7 ^a^ ± 2.4	41.4 ^a^ ± 1.1	43.1 ^a^ ± 1.0	42.1 ^a^ ± 2.0	43.2 ^a^ ± 2.1	42.7 ^a^ ± 1.3	43.0 ^a^ ± 2.2	44.3 ^a^ ± 1.0
BL (%)	8.0 ^b^ ± 2.1	9.2 ^b^ ± 1.1	7.0 ^b^ ± 1.2	6.3 ^b^ ± 3.0	7.2 ^b^ ± 2.0	7.5 ^b^ ± 3.2	9.3 ^b^ ± 3.7	8.7 ^b^ ± 2.4	7.4 ^b^ ± 2.0	6.3 ^b^ ± 1.3	17.7 ^b^ ± 2.0	16.6 ^b^ ± 2.4
Vol. (mL)	1090.1 ^c^± 25.2	631.4 ^c^ ± 25.3	802.1 ^c^ ± 36.6	517.6 ^c^ ± 15.7	823.1 ^c^ ± 38.1	835.2 ^c^ ± 10.2	845.1 ^c^ ± 12.2	859.1 ^c^ ± 10.2	862.3 ^c^ ± 23.5	884.2 ^c^ ± 17.8	941.7 ^c^ ± 26.8	989.8 ^c^ ± 10.8
Spe. Vol. (mL/g)	2.1 ^d^ ± 1.0	1.0 ^d^ ± 0.5	1.1 ^d^ ± 0.0	1.0 ^d^ ± 0.1	1.3 ^d^ ± 0.2	1.4 ^d^ ± 0.1	1.4 ^d^ ± 0.0	1.5 ^d^ ± 0.3	1.6 ^d^ ± 0.4	1.8 ^d^ ± 0.2	1.9 ^d^ ± 0.1	2.0 ^d^ ± 0.2

MC is moisture content, BL is baking loss, and Spe. Vol. is specific volume. The different letters in the superscript denote that the values are significantly different from each other at the 95% confidence interval.

**Table 2:** Pore analysis of the GH bread with different gelling agents in reference to the standard bread.

Bread Type	Pore Class (mm^2^)				
	Small (0.10–2.00)	Somewhat Medium (2.01–3.00)	Medium (3.01–6.00)	A Little Bigger (6.00–10.00)	Large (10.00–11.00)
Std. Yeast + EW+ RF	82.8 ^a^ ± 1.0	6.6 ^b^ ± 1.0	8.7 ^c^ ± 1.0	1.1 ^d^ ± 0.0	0.5 ^d^ ± 0.0
Std. EW + RF	93.7 ^a^ ± 1.0	3.1 ^b^ ± 1.0	1.5 ^c^ ± 1.2	3.9 ^d^ ± 0.7	0.0 ^d^ ± 0.0
Std. AC + EW + RF	92.4 ^a^ ± 2.1	4.1 ^b^ ± 1.4	5.1 ^c^ ± 1.9	1.0 ^d^ ± 0.2	0.5 ^d^ ± 0.0
40 + AC	85.2 ^a^ ± 2.4	4.3 ^b^ ± 1.1	8.3 ^c^ ± 1.1	2.2 ^d^ ± 0.2	0.3 ^d^ ± 0.0
40 + RF + EW	85.7 ^a^ ± 2.9	4.4 ^b^ ± 2.7	9.3 ^c^ ± 1.4	2.4 ^d^ ± 1.1	1.2 ^d^ ± 0.0
40 + AC + RF + EW	88.0 ^a^ ± 2.2	7.7 ^b^ ± 1.1	10.7 ^c^ ± 1.2	3.1 ^d^ ± 1.0	1.3 ^d^ ± 0.0
60 + AC	89.4 ^a^ ± 2.0	4.5 ^b^ ± 1.2	8.8 ^c^ ± 1.7	2.7 ^d^ ± 1.0	1.4 ^d^ ± 0.0
60 + RF + EW	89.9 ^a^ ± 2.8	5.5 ^b^ ± 1.8	8.9 ^c^ ± 1.3	2.8 ^d^ ± 1.2	1.4 ^d^ ± 0.0
60 + AC + RF + EW	90.5 ^a^ ± 2.1	6.6 ^b^ ± 1.9	8.9 ^c^ ± 2.4	3.1 ^d^ ± 1.3	1.5 ^d^ ± 0.0
70 + AC	89.8 ^a^ ± 2.9	5.7 ^b^ ± 2.3	8.9 ^c^ ± 1.7	2.9 ^d^ ± 1.0	1.5 ^d^ ± 0.0
70 + RF + EW	90.4 ^a^ ± 2.1	6.0 ^b^ ± 1.7	8.8 ^c^ ± 2.4	3.2 ^d^ ± 1.0	1.7 ^d^ ± 0.0
70 + AC + RF + EW	91.9 ^a^ ± 2.9	6.8 ^b^ ± 2.8	9.6 ^c^ ± 2.3	4.2 ^d^ ± 2.2	1.9 ^d^ ± 0.0

The different letters in superscript denote that the values are significantly different from each other at 95% confidence interval.

## Data Availability

The related data is included in the manuscript.
